# Disease of the Heart and Vascular System

**Published:** 1894-11-03

**Authors:** 


### Medical Progress and Hospital Clinics.

[*The Editor will be glad to receive offers of co-operation and contributions from members of the profession. All letters should be addressed to* The Editor, The Lodge, Porchester Square, London, W.]

### DISEASE OF THE HEART AND VASCULAR SYSTEM.—(*Continued.*)

### Valvular Lesions.

**Mitral Stenosis.**—The significance of the funnel-shaped and button-hole openings in stenosis of the cardiac valves has recently been discussed by Dr. Harry Campbell.[1] He called attention to the fact that physical investigations laws have demonstrated that the exit of fluid through an orifice is facilitated by a funnel-shaped passage leading to the orifice rather than by a mere circular orifice in a diaphragm which is flat, or in a mere partition, thus a funnel-shaped orifice, which is so usually found in valvular stenosis, has the effect of "making the best of a bad state of affairs."

*Treatment.*—Jas. Barr,[2] who has entered very fully into the treatment of mitral stenosis in former papers, contributes a valuable article on the various points to which our attention should be directed in overcoming the mechanical difficulties to the efficient working of the heart. In the early stages, when the valvular constriction is not very marked, a relatively slight hypertrophy of the ventricle may be sufficient to maintain the cardiac balance. We should then lessen the arterial tension which is usually present, so as to obviate all strain on the mitral valve, and thus prevent the development of the stenosis. The tension is best lessened by the administration of alkalies and salines, moderate exercise, a light diet with little fluid, and as in these cases there is generally a rheumatic tendency, the mode of life should be prophylactic against the development of acute attacks of rheumatism—warm clothing, morning cold bathing, and plenty of open-air exercise. The patient should be enjoined to "pursue the even tenor of his way" in a happy and contented frame of mind. Marriage cannot be recommended; and especially in the case of a female a timely counsel against wedlock and its usual results will be advisable.

Short of actual fatigue, a fair amount of exercise, even to the extent of climbing hills, will prove highly beneficial, by favouring the circulation, increasing the combustion, and improving the general nutrition, Excessive amount of fluid should be prohibited, and the regular use of alcoholic drinks should be strictly interdicted. Tea, coffee, and cocoa may be left to the discretion of the patient, except so far as quantity of fluid is concerned. Tobacco should be forbidden. In this early stage of the disease the use of drugs is unnecessary. If these rules be adhered to, the disease may make slow progress, and the patient enjoy many years of a very comfortable life, but the lesion has a progressive tendency.

When any indications of failure of compensation in the right side of the heart occur, there must be no fashionable treatment by graduated exercise on a mountain side, but the excellent old-fashioned restorative of rest in bed must be at once adopted. If the disease be in an early stage, a few days rest, a dry diet, or a cholagogue cathartic may be all that are required to restore the *status quo ante*, but if a long stay in bed be considered advisable, massage should be substituted for the loss of physical exercise. Dr. Barr has shown that the more or less constant high tension, observable in all cases of mitral stenosis, tends to lead to atheroma, and the only marked atheroma in the pulmonary veins that Dr. Barr has ever observed has been in cases of mitral stenosis. There is a tendency to pulmonary engorgement, which in course of time leads to brown induration of the lungs, excessive bronchial secretion, &c., and these conditions are best obviated by reducing the amount of fluid in circulation. These conditions are obtained by brisk catharsis, unless the case is very urgent, and then the right side of the heart may be relieved by venesection or by aspiration of the right side of the heart. When there is obvious failure in the force of the circulation, and the tricuspid is incompetent, with general engorgement of the whole venous circulation, resort to blood-letting must be interdicted. You only thus succeed in helping nature to empty an already depleted arterial system, and so hasten the not far-distant end; to this an exception must be made in cases of extreme urgency. In less urgent cases Dr. Barr would freely aspirate the liver or open the hæmorrhoidal veins. The right ventricle should be further relieved by brisk cholagogue cathartics. The action of the heart should be aroused by external warmth, sinapisms to the precordia, &c., and the internal use of such remedies as nitro-glycerine with atropine, ammonia, ether, or alcohol, these drugs to be given with as little fluid as possible; and when the patient is pulseless, ether and atropine may be injected hypodermically. When the urgent symptoms have been tided over, it may be necessary to give such cardiac remedies as digitalis, caffein, convallaria, and strophanthus. It is then more than ever necessary to insist on the general principles of diet and abstinence from liquids. When failure of the right ventricle with tricuspid regurgitation takes place the arterial pressure falls, the inward current of the blood is diminished, and the urine correspondingly fails. It is necessary then to improve the condition of the right side of the heart; diuresis is evidence of improved circulation, and shows that the cardiac tonics exhibited are having a good effect. While blood-letting may be of inestimable service in many cases, "if physicians could only be taught to lessen the amount of liquid ingested in cases where the heart is over-burthened with fluid, then venesection might often be dispensed with." The author concludes with a note on nitro-glycerine and atropine, regarding which he says that he always prescribes them in small doses—$\frac{1}{200}$ to $\frac{1}{50}$ of a minim of the former and $\frac{1}{200}$ to $\frac{1}{100}$ of a grain of the latter—sometimes separately and sometimes together, and the frequency of the dose is decided by the effects produced. In mitral stenosis it is not advisable to reduce the peripheral resistance too low, lest you produce worse effects than those one is trying to remedy.

**Mitral Regurgitation.**—The use of theobromine is advocated by Hallspear,[3] who has obtained good results with this drug in the case of a woman with mitral disease of many years standing, who presented œdema of the legs which resisted all other treatment. The medicine was prescribed in doses of fifty grains every six hours. She was so much relieved by the first two doses that after four the amount was reduced to three grains every twelve hours. The drug is supposed to have a greater effect on the heart and vessels than on the kidneys, and in this case the œdema was reduced very slowly.

[1] Lancet, Feb. 10, 1894. [2] Therap. Gazette. [3] Union Med., Jan. 30, 1894; Brit. Med. Journal Supplement, Vol. I., p. 32, 1894.

